# Phase I/II Clinical Trial of Encapsulated, Cytochrome P450 Expressing Cells as Local Activators of Cyclophosphamide to Treat Spontaneous Canine Tumours

**DOI:** 10.1371/journal.pone.0102061

**Published:** 2014-07-16

**Authors:** Monika Michałowska, Stanislaw Winiarczyk, Łukasz Adaszek, Wojciech Łopuszyński, Zbigniew Grądzki, Brian Salmons, Walter H. Günzburg

**Affiliations:** 1 Department of Epizootiology and Clinic of Infectious Diseases, Faculty of Veterinary Medicine, University of Life Sciences, Lublin, Poland; 2 Department of Pathological Anatomy, Faculty of Veterinary Medicine, University of Life Sciences Lublin, Lublin, Poland; 3 Austrianova, Centros, Singapore; 4 Department of Pathobiology, Institute of Virology, University of Veterinary Medicine, Vienna, Austria; University Hospital of Navarra, Spain

## Abstract

Based upon promising preclinical studies, a clinical trial was performed in which encapsulated cells overexpressing cytochrome P450 enzyme isoform 2B1 were implanted around malignant mammary tumours arising spontaneously in dogs. The dogs were then given cyclophosphamide, one of the standard chemotherapeutic agents used for the treatment of mammary tumours. The dogs were assessed for a number of clinical parameters as well as for reduction in tumour size. The treatment was well tolerated with no evidence of adverse reactions or side effects being associated with the administration of the encapsulated cells. Reductions in tumour size of more than 50% were observed for 6 out of the 11 tumours analysed while 5 tumours showing minor responses, i.e. stable disease. In contrast, the tumours that received cyclophosphamide alone showed only stable disease. Taken together, this data suggests that encapsulated cytochrome P450 expressing cells combined with chemotherapy may be useful in the local treatment of a number of dog mammary tumours and support the performance of further clinical studies to evaluate this new treatment.

## Introduction

The development and pathological features of spontaneously occurring mammary tumours in dogs are remarkably similar to breast cancer in women [1]–[3]. Indeed, the same cytological criteria that are applied in human pathology can be applied for the diagnosis of canine mammary gland tumours [4]. The incidence of these neoplasms in dogs is relatively high [5], [6], and, according to recent estimates, it is the most frequently diagnosed neoplasm in female dogs, accounting for 70% of all cancer cases [7]. Mammary tumours in dogs, like those in humans, are initially hormone-dependent and malignancy correlates with a loss of hormone responsiveness [8], [9]. Usually, primary mammary tumours are surgically resected. It has been reported that 58% of dogs with mammary cancer develop a new tumour after the surgical removal of the first tumour [10].

Cyclophosphamide is a standard chemotherapeutic agent that is commonly used to treat breast cancer in women [11] and has been used for this purpose also in dogs [2], [12]. Successful treatment is limited by relatively low concentrations of active anti-tumour metabolites reaching the tumour, due to the necessity for activation of the prodrug cyclophosphamide by cytochrome P450 enzymes expressed in the liver, followed by distribution of the active drug via the blood system [13], [14].

We have developed a system for the local conversion of chemotherapeutic agents such as cyclophosphamide (and the related agent ifosfamide) to their toxic metabolites, rather than the usual conversion that occurs primarily in the liver [14]–[16]. This system consists of cells that have been genetically modified to over express the cytochrome P450 gene. The cells are both immunoprotected and localised by encapsulation in polymers of cellulose sulphate [17], [18]. The cells containing the capsules are implanted either directly into the tumour or in the immediate area around the tumour. After implantation of the encapsulated cells, ifosfamide or cyclophosphamide is applied systemically. In this way elevated levels of the activated toxic prodrug metabolites are produced directly at the tumour site [19].

The DNA of all cells including the cells in the capsule are alkylated by the activated toxic prodrug metabolite phosphoramide mustard, but the cells are only killed when they try to divide. The cells in the capsule, and also non-tumour cells, are not dividing so they are not killed. We have shown that forced growth arrest of non-encapsulated cells indeed protects them from a self-killing effect in vitro [20]. We have also characterised the mechanism of cell killing [21].

Promising data have been obtained from this treatment protocol in both animal models of pancreatic adenocarcinoma [15] and in a human phase I/II clinical trial where 4 out of 14 patients with pancreatic cancer showed reductions in tumour size while the other 10 patients showed stable disease as well as a considerable survival benefit [16], [22]. Encouraging data using a similar strategy has also been obtained in two immunocompetent mouse models of mammary cancer [14], [23]. Based upon this data, a clinical protocol was developed for the treatment of spontaneously occurring malignant mammary tumours in dogs [24].

The current paper for the first time presents the primary data from the clinical protocol that was previously published [24]. The aim of the study was to investigate the effect of injecting cytochrome P450 overexpressing encapsulated cells around mammary tumours in dogs, followed by parenteral chemotherapy, on the cytoreduction of malignant tumour before surgery.

## Materials and Methods

### 1.1. Dogs and tumours

A total of 16 female dogs were enrolled in the study. The dogs were divided into two groups: a study group and a control group. The control group included 6 female dogs marked as: I, II, III, IV, V, and VI. The study group included 10 female dogs marked as: VII, VIII, IX, X, XI, XII, XIII, XIV, XV, and XVI. The mean age of the dogs that took part in the clinical trial was 8.8 years (range: 5–14 years) and the median age was 9 years. Due to the different breed of dogs included in the study, the body weight varied in the range: 9–43 kg with a mean of 18.8 kg and median of 17.8 kg. None of the 16 dogs included in the trial were spayed. Although most of the dogs (n = 13) had only a single detectable mammary tumour, four animals (IV, IX, XII, and XIII) had two tumours. Three of these dogs (IX, XII and XIII) were in the study group; whereas the dog marked IV was included in the control group.

The suitability of the animals for testing was based on the following criteria: 1) a cytological diagnosis confirming malignancy, 2) mammary tumour sizes being not less than 1 cm^3^ and not more than 250 cm^3^, 3) absence of regional metastatic lymphadenopathy, 4) absence of radiographic and ultrasonographic signs compatible with metastatic spread. The studies were reviewed and approved by the Ethics Committee of the University of Agriculture in Lublin (Poland) (Permission Number 53/2004).

### 1.2. Cells, capsule production and administration

A cell clone of the human embryonic kidney cell line, 293, transfected with a cytochrome P450 2B1 expression construct was used for encapsulation (CapCell) as previously described and characterised [16], [20], [25]. Expression of cytochrome P450 2B1 enables cells to convert chemotherapeutic agents like cyclophosphamide and ifosfamide to the tumour toxic metabolite phosphoramide mustard which causes DNA cross-linking. If a cell with damaged DNA tries to divide it is killed and this is the basis for the tumour selectivity of these chemotherapeutic agents [13], [15].

Encapsulation of the cells was achieved as previously described [17], [18] under “Good Manufacturing Practice (GMP)” conditions. Each dog received 20 cell-filled capsules per cm^3^ tumour, and the encapsulated cells were injected at five sites around the tumour using a G17 butterfly needle and 2 ml syringe [24]. Injections of the encapsulated cells typically required 5–10 minutes.

Although the protocol stipulated that the encapsulated cells should be applied to only one tumour in those animals with two or more tumours, the owner of one dog (XII) only agreed to the treatment if both tumours received the encapsulated cells. The other dogs with more than one tumour and not belonging to the control group (IX, XIII) were only treated with encapsulated cells at one tumour site, in line with the clinical trial protocol [24].

### 1.3. Chemotherapy

**Figure 1:**
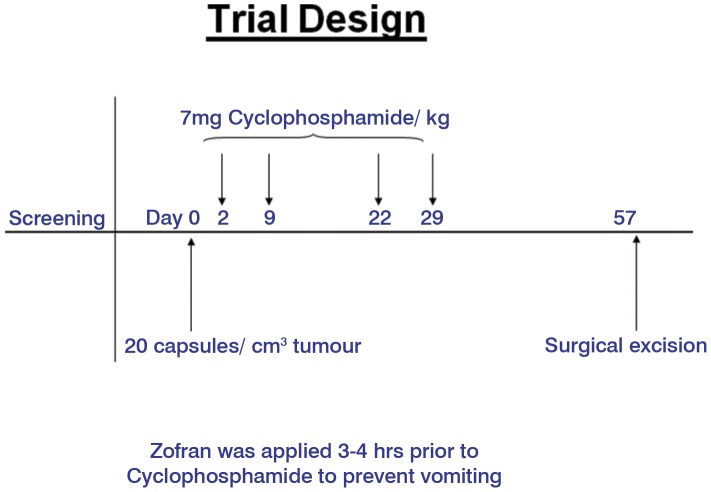
Design of clinical trial.

The dosage and administration schedule for cyclophosphamide was based on the scheme proposed by Löscher and co-workers [26]. Cyclophosphamide was obtained as a powder (200 mg per vial, Asta Medica) and solubilised in physiological saline at a concentration of 13.3 mg/ml just prior to infusion. A total of 4 infusions of cyclophosphamide, each at a concentration of 7 mg/kg body weight, were given by intravenous infusion. The first infusion was given on day 2 after instillation of the capsules in order to allow for an observation period in case there was an acute reaction to the encapsulated cells, and the subsequent 3 infusions were given on days 9, 22 and 29 following capsule administration (Tables 1 and 2, Fig. 1). Dog IX only received 79% of the dose of cyclophosphamide (50 mg) because it was 14 years old and the owner denied permission for full chemotherapy. The anti-nausea agent Zofran (GlaxoWelcome) was obtained as a ready-to-use solution and was given 3–4 hours before administration of cyclophosphamide at a concentration of 4 mg/dog (Fig. 1).

**Table 1:** Number of implanted CapCell and dosage of cyclophosphamide in the study group.

Female dog	Weight (kg)	Number of CapCell (day 0)	Cyclophosphamide (mg/dog)			
			day 2	day 9	day 22	day 29
**VII**	28	90	196	195	195	195
**VIII**	21	200	147	147	147	147
**IX**	9	750	50	50	50	50
**X**	12	140	87	87.5	84.7	79.8
**XI**	19.5	120	137	139	139	138
**XII**	12	200 and 320	84	84	84	77
**XIII**	11	94	77	77	84	84
**XIV**	30	450	210	210	210	210
**XV**	22	40	154	154	154	175
**XVI**	5.5	90	39	38	38	38

**Table 2:** Dosage of cyclophosphamide in the control group.

Female dog	Weight (kg)	Cyclophosphamide (mg/dog)				
		day 2	day 9	day 22	day 29	
**I**	20	140	140	140	140	
**II**	40	280	280	280	280	
**III**	40	280	280	280	280	
**IV**	16	112	112	105	105	
**V**	43	301	301	294	301	
**VI**	11	77	77	84	84	
**VII**	18	126	126	126	126	

### 1.4. Tumour measurement

Measurements of tumour dimensions were performed with a ruler and the largest three dimensional extensions of the tumour were used to calculate the tumour volume in cm^3^. Measurements were taken before treatment on the day of implanting encapsulated cells (baseline) and on days 9, 22, 29 and 57.

### 1.5. Criteria for the assessment of treatment efficacy and safety of the treatment

The general and preliminary criteria used to assess the safety of the treatment were haematological investigation, blood chemistry and urinalysis. The haematological investigation included determination of haemoglobin level, leucocyte and trombocyte count. Blood chemistry comprised determination of aspartate aminotransferase (AST), alanine aminotransferase (ALT) activity and blood urea nitrogen (BUN), creatinine, bilirubin and total protein levels. Urine was analysed by means of strips and, if necessary, confirmed by routine methods.

The final result of the treatment efficacy was evaluated based on the dynamics of tumour size change according to one of the following categories: 1. Complete remission (CR), 2. Partial remission (PR) – regression of the cancer by at least 50% of the initial tumour mass, 3. Stabilization of the disease (SD) – regression of the tumour by less than 50% or its enlargement by less than 25% of the initial tumour mass, 4. Progression of the disease (PD) – increase of the tumour size by at least 25% or new neoplastic foci.

### 1.6. Biopsies and histology

Biopsies, using a 0.9 mm diameter needle with a 20 ml syringe, were obtained from several sites from all tumours (in dogs from the study group, as well as control group) and transferred to slides. The cell smear was immediately fixed with Fixocyt (Polish Chemical Reagents, Gliwice) and stained with haematoxylin and eosin (H&E) and Papanicolaou stain. Resected tumour samples (on 57 days of the trial) destined for histopathology were fixed in 10% neutral buffered formalin, followed by embedding in paraffin. Sections 5 µm thick were prepared and stained with H&E. Tumours were classified after routine histopathological examination according to the WHO classification system [27].

## Results

10 dogs (13 tumours) formed the study group and were treated with both capsules and cyclophosphamide.

6 dogs formed the control group and were treated only with cyclophosphamide. The control group included additionally tumours in the XIII, and IX dogs without the CapCell implantation, considered an internal control site for the other tumour in these females; these tumours were taken into consideration in assessing treatment efficiency.

### 1.7. Safety of the treatment

Each dog in the study group received injections of the encapsulated cells around the tumour. The peri-tumour injections and the administration of the capsules were well tolerated, with no study medication related serious adverse events being observed in any of the dogs treated with the encapsulated cells over the entire observation period (56 days).

After administration of the encapsulated cells, the dogs received cyclophosphamide at a dose of 7 mg/kg. Some of the dogs (VII, IX, XI, XV) showed reduced appetite for 2–3 days after infusion of cyclophosphamide, a common side-effect of treatment with this chemotherapeutic agent [28]. The results of haematological examination of all dogs revealed mild thrombocytopenia (163–197×10^9^/L [reference ranges 200–500×10^9^/L]). In the following female dogs: V, VI, IX, XI, XII, and XVI a lower platelet count was found during the 2^nd^ and 3rd visit, and in female dogs: III, VIII, XIII, XIV and XV during the 2^nd^, 3^rd^ and 4^th^ visit. In 5 female dogs (I, II, IV, VII, and X) thrombocytopenia persisted throughout the therapy.

Some animals (XII, II, IV, IX and XV) showed urine abnormalities such as the presence of erythrocytes and/or traces of protein. Serum creatinine and BUN levels were slightly elevated in one dog, XV (creatinine 190.0 µmol/l -reference ranges 88,4–150,3 µmol/l, BUN 16.3 µmol/l -reference ranges 2,5–9,6 µmol/L, and the BUN level was increased for IX (18.8 µMol/L). AST levels rose in 6 to 10 hours and remained above the physiological range (1–37 U) for about 4 days in all dogs of the study group. The activity of ALT was also slightly elevated in most animals (reference ranges 4–91 U), although in all cases the abnormalities for all of these laboratory parameters subsided within a few days after treatment, and were self resolving.

Thus, in summary, the application of encapsulated cells in these 10 dogs (13 tumours) was well tolerated with no obvious side-effects. The subsequent treatment with cyclophosphamide resulted in mild side effects including thrombocytopenia which has been described previously as a side effect of cyclophosphamide treatment in dogs [29], [30], although these side effects were not exacerbated by the additional application of encapsulated cells.

### 1.8. Evaluation of tumour response/clinical efficacy

An evaluation of the tumour response was made for all of the 16 dogs (10 study group and 6 control group) participating in this clinical trial. The baseline tumour measurement (i.e. before treatment) ranged from 1.0 cm^3^ to 244.8 cm^3^ (Tables 3 and 4) with a median size of 6.6 cm^3^. Each tumour size at baseline was set as 100% and the size of the tumour after treatment given as a percentage of the baseline value. A total of 6 of the 11 evaluable tumours treated with encapsulated cells and cyclophosphamide showed a partial response, as characterised by a tumour regression of about 50%. The other 5 tumours in dogs given encapsulated cells and cyclophosphamide showed stable disease (i.e. a reduction in tumour size of less than 50% or no further tumour growth) (Table 5). This data contrasts with that obtained from tumours of dogs receiving only cyclophosphamide, where 6 tumours examined showed stable disease and 1 tumour showed progression of the disease (Table 6). The largest reduction in tumour size occurred during the 4 week treatment period for both groups, although clearly treatment with the encapsulated cells and cyclophosphamide resulted in a more pronounced tumour reduction, with a median reduction of 51.3% (6,7–70,2%) as compared to 21% (0–28,8%) after treatment with cyclophosphamide alone.

**Table 3:** Dynamics of tumour size changes in the study group.

Female dog	Location of tumour	Tumour size (cm^3^)				
		Initial	Visit 3	Visit 4	Visit 5	Visit 6
VII	R5	4.5	3.9	3.8	3.8	4.2
VIII	R3	1.0	1.0	0.9	0.7	0.7
IX	R3	37.1	34.7	28.9	28.9	28.9
X	R2	6.9	4.5	3.5	3	4
XI	L5	6.1	7.9	6.1	4.6	3.9
XII	L3	10	7.5	6.1	4.9	3.8
	L6	15.8	12.2	9.8	7.2	5.6
XIII	R4	4.7	2.7	2.5	2.5	1.4
XIV	R3	45	34.5	30.6	25.4	16.5
XV	L3	2.3	2.3	1.6	1.2	1.2
XVI	L5	4.5	2.8	1.2	1	1.5

**Table 4:** Therapeutic efficiency of general administration of cyclophosphamide in the control group.

Female dog	Location of tumour	Tumour size. cm^3^				
		Initial	Visit 3	Visit 4	Visit 5	Visit 6
I	L2	6.3	6.3	6	5	7.5
II	L4	244.8	242.2	221.3	203.4	174.2
III	R4	1.4	1.3	1.2	1.2	1.1
IV	L5	20.6	20.6	18.6	17.9	17.3
V	L5	4.5	4.3	3.6	3.6	3.6
VI	L4	15	15	15	13.8	13.2
XIII	R5	196	196	197	198	168

**Table 5:** Dynamics of tumour size change in the study group expressed as a percentage of initial tumour size.

Female dog	Location of tumour	Tumour size as % of initial size			
		Visit 3	Visit 4	Visit 5	Visit 6
VII	R5	86.7	84.4	84.4	93.3
VIII	R3	100	90	70	70
IX	R3	93.5	77.8	77.8	77.8
X	R2	65.2	50.7	43.5	43.5
XI	L5	24.6	100	75.4	64
XII	L3	75	61	49	38
	L6	77.2	62	45.6	35.4
XIII	R4	57.4	53.2	53.2	29.8
XIV	R3	76.7	68	56.4	36.7
XV	L3	100	69.6	52.2	52.2
XVI	L5	62.2	26.7	22.2	33.3

**Table 6:** Dynamics of tumour size change in the control group (K) expressed as a percentage of initial tumour size.

Female dog	Location of tumour	Tumour size as % of initial size			
		Visit 3	Visit 4	Visit 5	Visit 6
I	L2	100	95.2	79.4	119
II	L4	98.9	90	83.1	71.2
III	R4	92.9	85.7	85.7	78.6
IV	L5	95.6	80	80	80
V	L5	100	90.3	86.9	84
VI	L4	100	100	92	88
XIII	R5	100	100	100	85.7

More than one tumour was present in three dogs from the study treatment group (IX, XII, and XIII). Both tumours from dog XII were treated with encapsulated cells followed by cyclophosphamide and, interestingly, both tumours showed a similar reduction in tumour size (62 and 65%). It is also worthy of note that the encapsulated cell treated XIII tumour showed a reduction in size of 70%, whereas the second tumour in this dog, which did not receive encapsulated cells, only showed a 14% reduction, comparable to the reduction of 19% in tumour size in dogs from the control group.

### 1.9. Cytological examination

**Figure 2:**
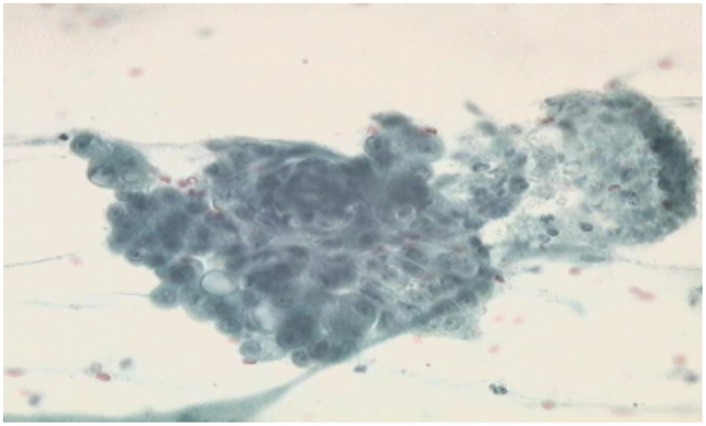
Results of cytology in female dog X, Papanicolaou staining, mag, x 400.

**Figure 3:**
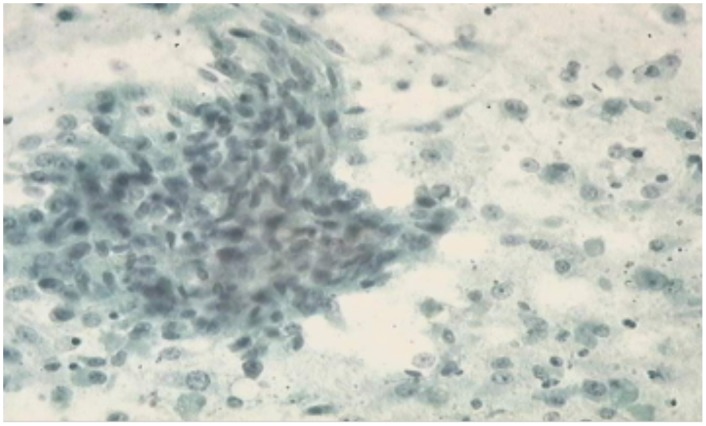
Results of cytology in XIII female dog Papanicolaou staining staining, mag, x 400.

In each of the 16 female dogs that qualified for inclusion in the study, the cytological picture of biopsy specimens obtained from all the tumours indicated malignancy of the neoplastic process. At the microscopic level, the most frequently registered were the presence of necrotic masses, neutrophilic granulocytes, deformed epithelial cells and epithelial cells with a varying degree of nuclear atypia (Fig. 2, 3).

### 1.10. Histological analysis and resection

Twenty tumours (13 tumours from 10 dogs in the study group, and 7 tumours from 6 dogs in the control group) were histopathologically tested.

Malignant tumours were found in all dogs. In 9 cases the diagnosis was complex carcinoma, in 4 cases simple tubulopapillary carcinoma, in 3 cases simple tubulopapillary carcinoma, partially solid, in 3 cases spindle cell carcinoma, and in one case the diagnosis was in situ carcinoma of the comedo type. An overview is given in Table 7.

**Table 7:** The histotypes of the tumors from female dog used in the study.

Hystotype	Number of cases	Female dog
complex carcinoma	9	I, IX (2 tumours), X, XII (2 tumours), XIII (2 tumours), XVI
simple tubulopapillary carcinoma	4	IV (2 tumours), VII, XI,
simple tubulopapillary carcinoma, partially solid	3	V, VI, XV,
spindle cell carcinoma	3	II, III, VIII
in situ carcinoma comedo type	1	XIV

**Figure 4:**
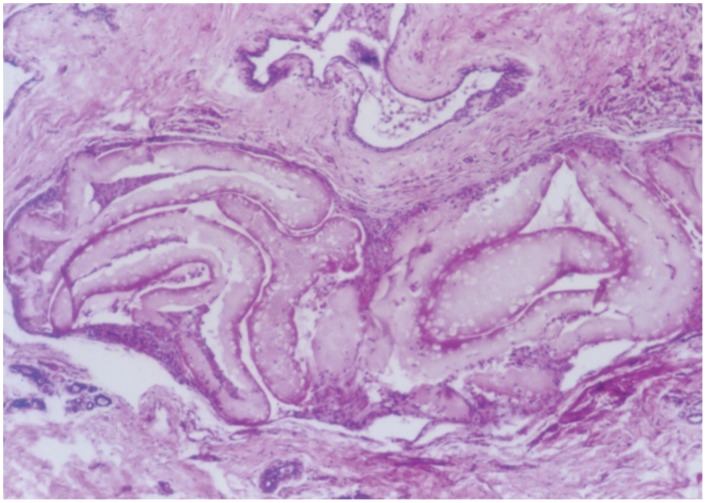
Section through the tumour and capsules after resection. Capsules, which have been deformed into elongated shapes by the sectioning, and the outer layer of tumour tissue are visible.

**Figure 5:**
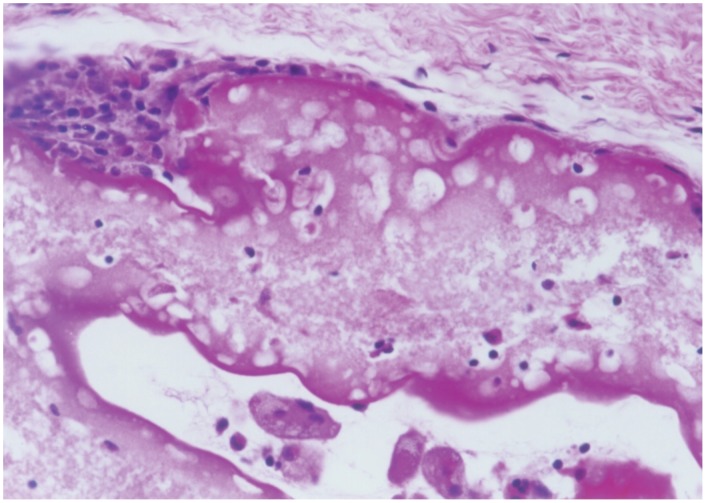
Close up of a capsule, revealing living HEK293 cells inside.

The encapsulated cells could be found in the tissue surrounding the resected tumours. This tissue did not show any evidence of an inflammatory reaction, although there was a slight fibrosis as demonstrated by the presence of fibroblasts and thin fibres of connective tissue. A weak infiltration of monocytes and macrophages could be observed at the border between a group of cell filled capsules and the surrounding tissue (Fig 4). Thus, these data suggest that the encapsulated cells were well tolerated and relatively inert, thus effectively shielding the cells inside the capsule from the host's immune system. Live 293 cells could also be seen within the capsules, suggesting that the cells were non-dividing since they were not killed by the toxic metabolites of cyclophosphamide (Fig 5).

## Discussion

In this study, the safety of introducing encapsulated cells capable of converting the chemotherapeutic agent, cyclophosphamide, to tumour toxic metabolites was examined. A secondary objective was to determine whether this local conversion had a better therapeutic effect than the usual cyclophosphamide treatment that relies on conversion of the chemotherapeutic agent by cytochrome P450 enzymes expressed in the liver.

Although side effects of the treatment could be observed, none of these were due to the implantation of encapsulated cells but rather to the administration of cyclophosphamide at the standard dose, since they were also experienced by the control animals receiving cyclophosphamide only. Histological sections of material resected from the tumour and surrounding areas showed that the encapsulated cells were well tolerated, with no or only mild immune reactions. These findings are in line with those from previous studies, both from a clinical phase I/II trial for pancreatic cancer in human patients [16], [31], [32], as well as in animal models [14], [15], [19], [33]. In this respect, it is worth mentioning that even encapsulated hybridoma cells producing a monoclonal antibody [34], [35] or encapsulated retroviral vector producing cells [36] have been shown to be well tolerated after implantation into immunocompetent mice. Nevertheless, immune responses can result if xenogenic cells are released, for example by capsule rupturing [37].

In studies performed in patients with inoperable pancreatic cancer, the microcapsules were angiographically placed in the blood vessels leading to the tumour. In the mammary cancer study reported here the microcapsules were injected peri-tumorally. Since similar tumour shrinkages were observed in both studies, this argues strongly that the results observed in the human clinical trial were not caused by vessels being blocked by microcapsules, but rather from the focusing of the toxic effect of cyclophosphamide on the tumour. In follow-up studies, similarly to the study reported here, no allergic reactions or signs of pancreatitis were observed in these patients. These results demonstrate that microcapsules effectively protect the encapsulated cells against attack by the host's immune system whilst allowing free diffusion of nutrients from the external environment into, and the release of cellular metabolites out of, the microcapsule. [34], [35], [37], [38].

In this study, peri-tumoral injection of encapsulated cells, followed by systemic application of cyclophosphamide, was used in a neo-adjuvant setting to shrink tumours before they were surgically removed. This improves the success of the surgical excision since less skin has to be removed, hence improving wound closing and thus the healing process [39]. A long term benefit may be expected since a smaller tumour is generally associated with a better prognosis, even after surgical removal has taken place [2], [9]. It should also be possible to combine the encapsulated cell therapy with other treatments such as gemcitabine, which has recently been shown to give significantly improved overall survival rates, but only when given in an adjuvant setting after surgical resection [40]. Such a combination trial might be of interest since 58% of dogs with mammary cancer develop a new tumour after the surgical removal of the first tumour [10].

## Conclusion

The use of encapsulated drug converting cells for improved, local chemotherapy is a safe treatment strategy. The use of this strategy results in improved anti-tumour effects as measured by tumour reductions, suggesting that it may be useful for the treatment of mammary tumours in human and animal patients. Thus, further clinical studies are warranted.
